# Clinical evaluation of suture materials for transtibial pullout repair of medial meniscus posterior root tear

**DOI:** 10.1186/s43019-022-00167-x

**Published:** 2022-10-08

**Authors:** Takaaki Hiranaka, Takayuki Furumatsu, Yuki Okazaki, Keisuke Kintaka, Yusuke Kamatsuki, Ximing Zhang, Haowei Xue, Masanori Hamada, Toshifumi Ozaki

**Affiliations:** 1 Department of Orthopaedic Surgery, Okayama University Hospital, 2-5-1 Shikatacho, Kitaku, Okayama, 700-8558 Japan; 2 Department of Orthopaedic Surgery, Ako Central Hospital, 52-6 Soumoncho, Ako, Hyogo 678-0241 Japan; 3 Department of Orthopaedic Surgery, Okayama University Graduate School, 2-5-1 Shikatacho, Kitaku, Okayama, 700-8558 Japan

**Keywords:** Medial meniscus, Posterior root tear, Clinical outcome, Meniscal healing, Suture material, Pullout repair

## Abstract

**Background:**

There are no recommendations for specific suture materials in transtibial pullout repair of medial meniscus posterior root tears. This study aimed to evaluate the clinical outcomes of transtibial pullout repair of medial meniscus posterior root tears using ultrahigh-molecular-weight polyethylene sutures and suture tape.

**Methods:**

We retrospectively reviewed the data of 36 patients (27 women and 9 men, mean age 64.1 years) who had undergone transtibial pullout repair of medial meniscus posterior root tears between November 2018 and December 2019. Two groups of 18 patients each received either two different cord-like sutures or suture tape. Clinical parameters were assessed preoperatively and on second-look arthroscopy (mean postoperative period 12 months). The meniscal healing status was assessed using a previously published scoring system (ranging from 0 to 10), and the incidence rate of suture cut-out was assessed on second-look arthroscopy.

**Results:**

All clinical scores significantly improved in both groups, with no significant between-group differences on second-look arthroscopy. The arthroscopic meniscal healing scores significantly differed between sutures (mean 6.7 points) and suture tape (mean 7.4 points; *p* = 0.044). No significant between-group difference in the suture cut-out rate was observed.

**Conclusions:**

This study found no significant differences in the clinical outcomes between ultrahigh-molecular-weight polyethylene sutures and suture tape. Favorable clinical outcomes were obtained using both types of suture; however, the usefulness of suture tape appears to be limited.

**Supplementary Information:**

The online version contains supplementary material available at 10.1186/s43019-022-00167-x.

## Background

Medial meniscus (MM) posterior root tear (MMPRT) causes medial meniscal extrusion, which remarkably impairs hoop tension and load-sharing ability [1, 2]. Thus, untreated MMPRT may lead to rapid osteoarthritis progression in the knee joint’s medial compartment [3, 4]. As prior research has indicated that MMPRTs are biomechanically equivalent to total medial meniscectomy [5], accurate diagnosis and early repair are essential for subsequent treatment [6]. Several techniques have been introduced for MMPRT repair; transtibial pullout repair is frequently performed, as it does not require posterior portals, whereas trans-osseous drilling may enhance meniscal healing through stem-cell recruitment from the bone marrow [7, 8]. Various studies have evaluated biomechanical strength and clinical outcomes across different suture techniques [9, 10]; however, there is no recommended suture material for the transtibial pullout repair of meniscus root tears.

Various materials such as absorbable monofilament polydioxanone [9, 11, 12], braided non-absorbable polyester [13–15], and high-strength [composed of ultrahigh-molecular-weight polyethylene (UHMWPE)] sutures [16, 17] have been used during arthroscopic procedures. Suture material designs have evolved through the introduction of UHMWPE-incorporating polyblend sutures. Sutures containing UHMWPE are stronger and stiffer than conventional sutures, making them suitable for repairs. Additionally, they are available as both cord-like (UHMWPE suture) and tape-shaped (UHMWPE suture tape) sutures; a wider suture tape could fill the hole created for the passage of the suture through the tissue and ensure better distribution of forces to avoid suture cut-out [11, 18].

Although many studies have reported the biomechanical superiority of UHMWPE suture tape to UHMWPE sutures [19–22], to our knowledge, no prior studies have examined the clinical superiority between these suture materials. Therefore, this study aimed to compare the clinical outcomes between cases where UHMWPE sutures were used and those where UHMWPE suture tape was used, including assessment of the meniscal healing status and the rate of suture cut-out on second-look arthroscopy. We tested the hypothesis that the clinical outcomes associated with UHMWPE suture tape would be superior to those associated with UHMWPE sutures.

## Methods

### Patients

This study was approved by our institutional review board, and written informed consent was obtained from all patients prior to their participation per the principles embodied in the Declaration of Helsinki. In this study, a total of 36 patients (27 women and 9 men) who underwent transtibial pullout repair between November 2018 and December 2019 were retrospectively investigated. The following patients were included: those diagnosed with an MMPRT, those who met the operative indication (femorotibial angle < 180°, Outerbridge grade I or II, and Kellgren–Lawrence grades 0–2), and those who underwent pullout repair surgery. Patients with missing clinical or radiographic data, with previous histories of meniscus or knee surgery, or without a painful popping event were excluded. The patients were divided into two groups: one in which two different UHMWPE sutures were used (*n* = 18); these were either ULTRABRAID (*n* = 7; Smith & Nephew, Andover, MA, USA) or FiberWire (*n* = 11; Arthrex, Naples, FL, USA), and another in which UHMWPE suture tape (*n* = 18), namely ULTRATAPE (Smith & Nephew) (*n* = 18), was used. Eighteen patients in each group underwent transtibial pullout repair using either UHMWPE sutures between November 2018 and April 2019 or UHMWPE suture tape between May and December 2019. Second-look arthroscopy and screw removal were performed 12 months postoperatively in patients who desired these procedures.

### Surgical procedures

**Fig. 1:**
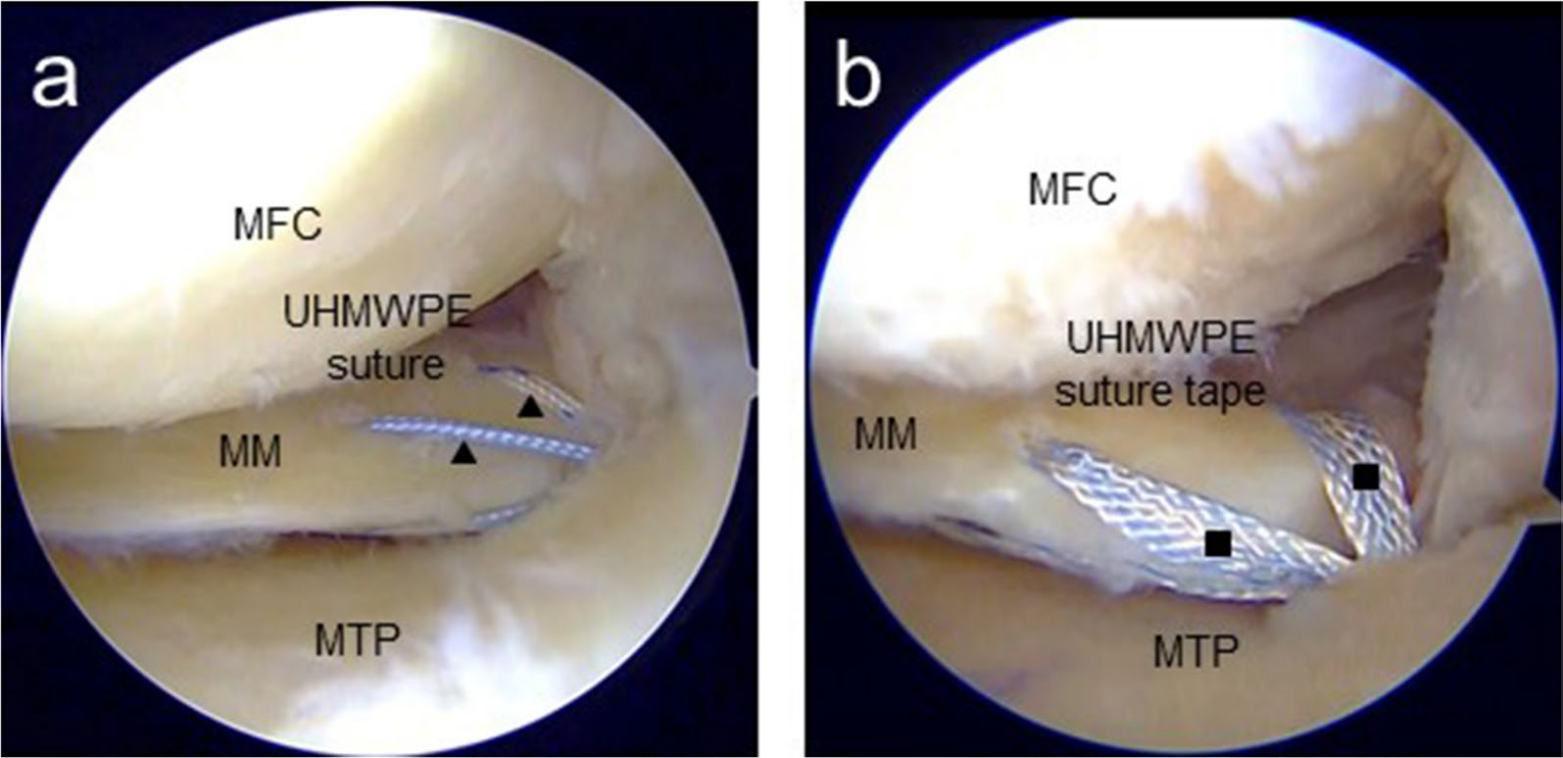
Arthroscopic findings after pullout repair (left knee). **a** Black triangles indicate UHMWPE sutures. **b** Black squares indicate UHMWPE suture tape. *MFC* medial femoral condyle; *MM* medial meniscus; *MTP* medial tibial plateau; *UHMWPE* ultrahigh-molecular-weight polyethylene

Standard arthroscopic examinations in both groups were performed using a 4-mm-diameter 30° arthroscope (Smith & Nephew). The outside-in pie-crusting of the medial collateral ligament was used to widen the medial compartment, and a Knee Scorpion suture passer was used to vertically pass two UHMWPE sutures or two UHMWPE suture tapes through the meniscal tissue. The first needle was inserted into the inner area of the posterior horn of the MM, and the second needle into the outer area of the posterior root of the MM, 10 and 4 mm from the torn area, respectively (Fig. 1). An all-inside suture device, such as the FasT-Fix repair system (Smith & Nephew), was inserted through the anterolateral portal. During this procedure, the first anchor of the device was inserted into the inferior surface of the MM posterior segment, whereas the second one was inserted directly into the articular capsule toward the posteromedial direction. A 4.0- or 4.5-mm tibial tunnel was created in the MM posterior root attachment using an MMPRT guide (Smith & Nephew) or Unicorn Meniscal Root guide (Arthrex). There were no cases of non-anatomic repair in this study. Tibial fixation was performed using a bioabsorbable interference screw and spring tensioner at the expected knee flexion (30°) and tension (20 N).

### Postoperative rehabilitation protocols

Weight-bearing on the knee immobilizer was not allowed for 2 weeks postoperatively. Knee flexion was limited to 90° for the first 4 weeks, and full weight bearing and 120° knee flexion were allowed after 6 weeks. Deep knee flexion was allowed 3 months postoperatively.

### Assessment methods

Clinical evaluations were performed during primary surgery and second-look arthroscopy. Clinical outcomes were evaluated using the Japanese Knee Injury and Osteoarthritis Outcome Score, Lysholm knee score, International Knee Documentation Committee subjective knee evaluation form, and visual analog scale pain score. The Japanese Knee Injury and Osteoarthritis Outcome Score comprises five subscales: pain, symptoms, activities of daily living, sport and recreation function, and knee-related quality of life. The pain intensity of the knee was assessed using a 100-mm visual analog scale, ranging from 0 (no pain) to 100 mm (worst possible pain).

### Arthroscopic meniscal healing scores

The healing status of the MM posterior root was evaluated during second-look arthroscopy according to a previously reported scoring system [23]. This semi-quantitative arthroscopic scoring system comprises three criteria for evaluation: anteroposterior width of bridging tissues, stability of the repaired MM posterior root, and synovial coverage of the sutures; scores range from 0 to 10 points. Regarding the anteroposterior width of bridging tissues, 4, 2, or 0 points would be assigned for broad (> 5 mm), narrow (2–5 mm), and filamentous (< 2 mm) bridging tissue, respectively. The stability of the repaired MM posterior root would be assigned a score of 4, 3, 2, 1, or 0 points, depending on the degree of lifting and anterior drawing of the meniscus root by probing. Regarding synovial coverage, suture coverage would be scored as good (2 points), fair (1 point), or poor (0 point), according to arthroscopic findings. In addition, two blinded orthopedic surgeons retrospectively evaluated the meniscal healing scores; the derived mean score for each patient was used in the subsequent analyses.

### Magnetic resonance imaging evaluation

Magnetic resonance imaging (MRI) evaluation was performed on an Achieva 1.5 T (Philips, Amsterdam, the Netherlands) using a knee coil, as previously reported. The standard sequence was T2-weighted fast-field echoes in the sagittal (TR/TE, 742/18), coronal (TR/TE, 637/18), and axial (TR/TE, 499/18) planes at a 20° flip angle. The slice thickness and gap were 3 and 0.6 mm, respectively. The field of view was either 16 or 17 cm, and the imaging matrix size was either 205 × 256 or 200 × 368. The meniscus was evaluated using proton density-weighted MRI because of its good visibility; MM extrusion (MME) was evaluated by MRI and measured from the inner edge of the tibial plateau to the outer edge of the MM. The presence of osteophytes in the medial tibial plateau was excluded when obtaining tibial MME. Pre- and postoperative MME was compared between the two groups.

### Statistical analysis

Statistical analyses were performed using EZR software (Saitama Medical Center, Jichi Medical University, Saitama, Japan). The Mann–Whitney *U* test was used to compare intergroup differences during primary surgery and second-look arthroscopy, whereas Fisher’s exact test was used to compare differences between the sexes and the suture cut-out; statistical significance was set at *p* < 0.05. The interobserver reproducibility and intraobserver repeatability of the meniscal healing score were considered high, with mean intraclass correlation coefficient values of 0.87 and 0.91, respectively.

## Results

Sixty-seven patients who underwent transtibial pullout repair for MMPRT were enrolled in this study. Twenty-two patients with incomplete clinical or radiographic data, two with previous histories of meniscus injury or knee surgery, and seven without painful popping were excluded. Ultimately, the data of 36 patients were retrospectively evaluated. There were no significant between-group differences concerning preoperative patient demographics (Table 1, Additional file 1: Fig. S1) and clinical scores (Table 2). All clinical scores significantly improved in both groups, and no significant between-group differences in clinical scores were observed 1 year postoperatively (Table 2). Additionally, a significant between-group difference in arthroscopic meniscal healing scores was identified between the two groups [6.7 points (mean) for UHMWPE sutures versus 7.4 points (mean) for UHMWPE suture tape; Table 2], and there was no significant between-group difference in any of the criteria (Table 2). Suture cut-out was observed in 14 and 12 patients using the UHMWPE sutures and UHMWPE suture tape, respectively (Table 2). No significant between-group differences were noted regarding the rate of suture cut-out. The postoperative knee flexion angle was 124.7 ± 4.5° and 123.6 ± 7.0° in the UHMWPE sutures and UHMWPE suture tape groups, respectively (*p* = 0.727). Besides, no significant between-group difference was observed regarding pre- and postoperative MME (Table 3).

**Table 1:** Patient demographics and clinical characteristics

	UHMWPE sutures	UHMWPE suture tape	*p* value
Number (knees)	18	18	
Sex (male/female)	7/11	2/16	0.121
Age (years)	63.1 ± 13.4	65.3 ± 8.7	0.428
Height (m)	1.59 ± 0.1	1.57 ± 0.1	0.170
Weight (kg)	64.1 ± 15.1	60.5 ± 8.9	0.234
Body mass index (kg/m^2^)	25.2 ± 3.3	24.5 ± 3.6	0.498
Duration from injury to operation (days)	74.7 ± 72.8	75.2 ± 53.2	0.309
Duration from primary surgery to second-look arthroscopy (days)	385.4 ± 21.6	381.1 ± 25.4	0.594
Femorotibial angle (°)	177.3 ± 1.8	177.6 ± 1.8	0.752
Root tear classification (type 1/2/3/4/5) [24]	0/14/0/4/0	0/14/0/4/0	1.000

Values are presented as mean ± standard deviation or number

*UHMWPE* ultrahigh-molecular-weight polyethylene

The arthroscopic view of type 2 and 4 root tear [24] was shown in Fig. S1

**Table 2:** Comparison of preoperative and 1-year postoperative clinical scores between UHMWPE sutures and UHMWPE suture tape

	Preoperative			Postoperative		
	UHMWPE sutures	UHMWPE suture tape	*p* value	UHMWPE sutures	UHMWPE suture tape	*p* value
KOOS, Pain	54.8 ± 15.3	59.1 ± 19.0	0.661	86.9 ± 10.9	85.2 ± 13.8	0.568
KOOS, Symptoms	56.3 ± 17.6	59.9 ± 21.6	0.596	74.9 ± 14.5	77.7 ± 13.0	0.609
KOOS, ADL	65.1 ± 18.2	66.0 ± 19.7	0.971	84.1 ± 9.4	86.6 ± 15.6	0.596
KOOS, Sport/Rec	21.4 ± 22.9	25.0 ± 27.1	0.849	45.3 ± 28.6	53.2 ± 28.6	0.334
KOOS, QOL	22.6 ± 16.4	38.0 ± 21.1	0.255	62.7 ± 15.2	65.1 ± 20.7	0.819
Lysholm knee score	55.3 ± 14.4	62.1 ± 10.1	0.344	85.6 ± 21.2	88.2 ± 7.7	0.909
IKDC score	37.1 ± 15.1	40.1 ± 16.1	0.791	60.2 ± 13.6	68.4 ± 10.5	0.151
Pain score (VAS)	40.0 ± 19.4	33.6 ± 27.7	0.546	13.1 ± 13.1	7.4 ± 11.3	0.344
Arthroscopic healing score^a^ (points)				6.7 ± 1.0	7.4 ± 1.7	0.044*
Anteroposterior width of bridging tissues (points)				3.8 ± 0.1	3.9 ± 0.1	0.540
Stability of the medial meniscus posterior root (points)				2.3 ± 0.1	2.6 ± 0.2	0.358
Synovial coverage of the sutures (points)				0.7 ± 0.1	0.9 ± 0.1	0.231
Suture cut-out (positive/negative)				14/4	12/6	0.488

Values are presented as mean ± standard deviation

*UHMWPE* ultrahigh-molecular-weight polyethylene; *KOOS* Knee Injury and Osteoarthritis Outcome Score; *ADL* activities of daily living; *Sport/Rec* sport and recreation function; *QOL* knee-related quality of life; *IKDC* International Knee Documentation Committee; *VAS* visual analog scale

*p < 0.05

^a^Arthroscopic healing score on second-look arthroscopy (total, 10 points)

**Table 3:** Comparison of preoperative and 1-year postoperative radiographic outcomes between UHMWPE sutures and UHMWPE suture tape

	Preoperative			Postoperative		
	UHMWPE sutures	UHMWPE suture tape	*p* value	UHMWPE sutures	UHMWPE suture tape	*p* value
Kellgren–Lawrence grade (0/1/2/3/4)	0/8/10/0/0	0/9/9/0/0	1.000	0/6/12/0/0	0/6/12/0/0	1.000
Medial meniscus extrusion (mm)	2.9 ± 1.1	3.0 ± 0.9	0.782	3.4 ± 1.2	3.6 ± 0.8	0.642

Values are presented as mean ± standard deviation

## Discussion

The key finding of this study was that favorable clinical outcomes of pullout repair were obtained with both UHMWPE sutures and UHMWPE suture tape; no significant differences were observed between the two suture materials, excluding the meniscal healing status.

Obtaining an anatomically healed and biomechanically functional meniscus after repair surgery is crucial, as the meniscus–suture interface is the most susceptible site to early failure. Therefore, the suture material must provide low displacement, high stiffness, and maximum tensile strength. These characteristics are required to keep the reattached meniscus root in place during the healing process. Sutures containing UHMWPE can absorb a large amount of energy and remain flexible [20]. Nevertheless, biomechanical studies have shown that UHMWPE exhibits higher load-to-failure or stiffness than conventional suture materials [25, 26]. Matthews et al. [20] compared the biomechanical properties of various suture materials under cyclic and load-to-failure conditions using porcine menisci and reported that the maximum load-to-failure of UHMWPE ranged from 124 to 287 N. For example, the maximum load-to-failure of No. 2 ULTRABRAID, 2-0 FiberWire, and ULTRATAPE used in this study were 218.9, 124.6, and 198.9 N, respectively. These loads were superior to the tensile forces (60 N) acting on the repaired MM posterior roots generated with internal rotation, a 500-N load, and 90° flexion [27]. Therefore, sutures containing UHMWPE may provide protective benefits during knee flexion and weight bearing and are thus suitable for meniscal repair.

Sutures containing UHMWPE come in either a cord-like or a suture tape form. The recently introduced suture tapes are increasingly used to repair degenerated tissues, such as rotator cuffs [28]. They have a broader surface area for load distribution, which may increase the load required for suture cut-out and reduce repair failure. Taha et al. [21] concluded that suture tape was superior to cord-like sutures, providing reduced creep, greater stiffness, and reduced extensibility during arthroscopic shoulder procedures. Burgess et al. [19] compared FiberTape (Arthrex) and FiberWire in vitro, demonstrating that FiberTape was stronger and stiffer. Robinson et al. [22] reported that repairs using 2-mm UHMWPE tape provided superior initial fixation strength compared with No. 2 sutures via a porcine model. On the basis of these studies, UHMWPE suture tape appeared to be the superior material for MMPRT repair, demonstrating better biomechanical properties than UHMWPE sutures.

Although many studies have compared the biomechanical properties of suture materials, to our knowledge, none has compared the clinical outcomes following meniscus repair using different suture materials. Herein, better meniscal healing was observed in cases where UHMWPE suture tape was used than in those where two different UHMWPE sutures were used. These findings are analogous to those of the cited studies that demonstrated the superior biomechanical properties of UHMWPE. Improved meniscal healing may contribute to the arrest of osteoarthritis progression, leading to good long-term clinical outcomes and explaining the superiority of UHMWPE suture tape [23].

This study obtained favorable clinical outcomes with both suture materials, comparable to the findings of other reports. This may indicate that the influence of differences in suture materials during meniscal healing might be trivial. There were also no significant differences in the incidence rate of suture cut-out on second-look arthroscopy. Further evaluation with larger sample sizes and longer follow-up periods is necessary to expand on our results. The cost of UHMWPE suture tape is higher than that of UHMWPE sutures (57,600 versus 3500–3650 yen); thus, the use of UHMWPE sutures could contribute to a reduction in the total medical cost of pullout repair. Although the usefulness of UHMWPE suture tape is limited, both suture types could be used for pullout repair of MMPRT.

This study has several limitations. First, the postoperative follow-up period was short, and the sample size was too small to evaluate and compare the clinical outcomes following MMPRT repair. Second, this was a retrospective study and may have involved selection bias. Finally, although two different UHMWPE sutures (ULTRABRAID and FiberWire) were used, they were not evaluated separately. The suture structures of these subtypes differ [20]; FiberWire is a non-absorbable polyester suture with a UHMWPE multifilament core and a braided polyester jacket, whereas ULTRABRAID contains braided, non-absorbable polyethylene fibers without a longitudinal core. Thus, biomechanical differences exist between these materials, which may be a strong limitation to this study. While there were no significant differences in the meniscal healing status between the two suture types [6.6 points (mean) for FiberWire versus 6.8 points (mean) for ULTRABRAID], the structural differences may have been a possible confounder affecting the results.

## Conclusions

There was no significant difference in the clinical outcomes of procedures performed using UHMWPE sutures or UHMWPE suture tape, and good clinical outcomes were obtained using both. However, the greater clinical usefulness of UHMWPE suture tape could not be proven; thus, larger, prospective studies with longer follow-up periods are necessary to compare the clinical outcomes with different suture types in MMPRT cases.

## Supplementary Information

** Additional file 1.** Arthroscopic view of type 2 (left) and type 4 MMPRT (right).

## Data Availability

The datasets generated during and/or analyzed during the current study are available from the corresponding author on reasonable request.
