# Supplementary figures and images for: Clinical evaluation of suture materials for transtibial pullout repair of medial meniscus posterior root tear
Source: Knee Surg Relat Res. 2022 Oct 8;34:39. doi: 10.1186/s43019-022-00167-x (PMC9548199; doi:10.1186/s43019-022-00167-x)

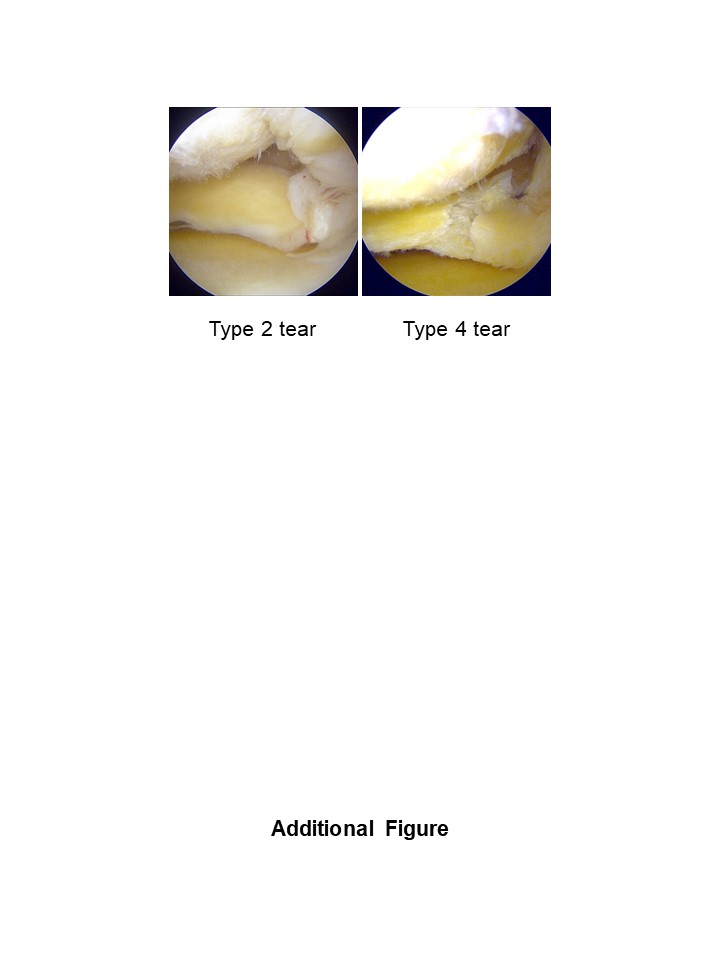

Supplement: Supplementary file 1 — Additional file 1. Arthroscopic view of type 2 (left) and type 4 MMPRT (right). [file 43019_2022_167_MOESM1_ESM.jpg]
